# Pre-ART nutritional status and its association with mortality in adult patients enrolled on ART at Fiche Hospital in North Shoa, Oromia region, Ethiopia: a retrospective cohort study

**DOI:** 10.1186/s13104-016-2313-y

**Published:** 2016-12-20

**Authors:** Kokeb Tesfamariam, Negga Baraki, Haji Kedir

**Affiliations:** 1 College of Medicine and Health Sciences, Department of Public Health, Ambo University, P.O. Box 21115, Ambo, Ethiopia; 2 School of Public Health, Haramaya University, Harar, Ethiopia

**Keywords:** Antiretroviral therapy, Malnutrition, Mortality

## Abstract

**Background:**

Human immunodeficiency virus (HIV) compromises the nutritional status of infected individuals and in turn, malnutrition worsens the effects of the infection itself by weakening the immune system consequently accelerating disease progression and death. However, few studies have examined the association between nutritional status at antiretroviral therapy (ART) initiation and early mortality. Therefore, this study assesses pre-ART nutritional status and other baseline characteristics and mortality among adult patients on ART at Fiche Hospital, Ethiopia.

**Methods:**

A retrospective cohort study was conducted among 489 ART enrolled adult patients between August 01, 2006 and September 30, 2013 in Fiche Hospital. Study participants were selected by using systematic random sampling method. Actuarial table was used to estimate survival of patients after ART initiation and log rank test was used to compare the survival curves. Cox proportional-hazard regression was used to determine independent predictors of time to death.

**Results:**

Most of the study subjects were females 254 (51.9%). A total of 489 patients were included in the analysis, of whom 87 died during a median study follow-up of 22 months. The estimated mortality among malnourished was 21, 28, 33, and 38% at 5, 10, 15, and 25 months respectively with mortality incidence density of 5.63 deaths per 100 person years. The independent predictors of mortality were: BMI <18.5 kg/m^2^ (AHR = 5.4 95% CI 3.03–9.58), baseline ambulatory functional status (AHR = 3.84; 95% CI 2.19–6.74), bedridden functional status (AHR = 4.78; 95% CI 2.14–10.65), WHO clinical stage III (AHR 2.21; 95% CI 1.16–4.21), WHO clinical stage IV (AHR 4.05; 95% CI 1.50–10.97) and CD4 count less than 200 cells/μl (AHR = 2.95; 95% CI 1.48–5.88), two and more opportunistic infections (AHR 2.30; 95% CI 1.11–4.75).

**Conclusions:**

Undernutrition at the time of ART initiation was associated with increased risk of death, particularly during the first 3 months after ART initiation. Interventions to promote earlier HIV diagnosis and treatment and integrating nutrition counseling at all stages of ART implementation may improve ART outcomes in this vulnerable population.

## Background

Provision of antiretroviral therapy (ART) for HIV-infected individuals is rapidly expanding in sub-Saharan Africa [1]. HIV itself increases resting energy expenditure independently of viral load, further contributing to HIV-associated weight loss. As HIV infection progresses, it can cause a catabolic state that is compounded by a lack of caloric intake, increasing the severity of preexisting undernutrition [2].

Body mass index (BMI) at ART initiation was defined as BMI below 18.5 kg/m^2^ indicates that a person is underweight, 18.5–24.9 kg/m^2^ is normal weight, and 25.0–29.9 kg/m^2^ is overweight and 30.0 kg/m^2^ or above is obese. HIV compromises the nutritional status of infected individuals and in turn, malnutrition worsens the effects of the infection itself by weakening the immune system consequently accelerating disease progression and death [3].

An improved understanding of the role of nutritional status in HIV disease progression may help in the development of strategies to reduce mortality after ART initiation. To the best of our knowledge, no previous study has examined the effect of pre-ART nutritional status on time to death in the cohort of ART clients enrolled during the specified time in the study area. This study, therefore, intends to examine the associations between nutritional status and its associated mortality among adult patients on ART. Moreover, this study results serve as baseline data for further investigations and provides input for health planners and policy makers.

## Methods

Institution based retrospective cohort study was conducted in Fiche Hospital from Jan 01–31/2014. Fiche town has one zonal hospital and two health centers for the catchment area population, which provide ART service for 3937 patients enrolled. Peoples’ living with HIV/AIDS (PLWHA).

### Inclusion criteria

HIV positive adults aged 18 years or older who started ART with complete intake form, registers that have been in follow-up from 2006 to 2013.

### Exclusion criteria

- Diagnosis is made outside of health institution (transfer in).
- Loss to follow up, transfer out.
- Pregnant and lactating women at the time of ART initiation.

### Sample size determination

Sample size was determined using a formula for two population proportions based on the assumption that type I error 5%, power of 90% on exposure (malnourished on pre-ART treatment) and non-exposure (non-malnourished on pre-ART treatment) was taken from previous study [15].

$${\text{n}}_{1} = \frac{{\left[ {Z_{{\frac{\alpha }{2}}} \sqrt {\left( {1 + \frac{1}{r}} \right)P\left( {1 - P} \right)} + Z_{\beta } \sqrt {P_{1} \left( {1 - P} \right)_{1} + \left( {\frac{{P_{2} \left( {1 - P_{2} } \right)}}{r}} \right)} } \right]^{2} }}{{\left( {P_{1} - P_{2} } \right)^{2} }}$$

α = level of significance, power = 1 − β = 90%, Z_β_ = 1.282.

The sample size was 163 for n_1_ (exposed group) and 326 for n_2_ (non-exposed group). Using proportional allocation to the malnourished and non-malnourished adult patients, a total of 489 samples taken.

### Sampling technique

**Fig. 1:**
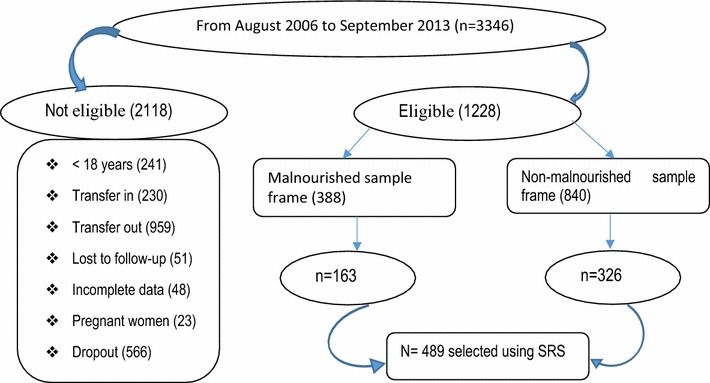
Profile of PLWHA enrolled on ART in Fiche Hospital, North Shoa, Ethiopia

A cohort of antiretroviral patients who were initiated treatment between August 2006 and September 2013 were included in the study and their profiles were evaluated. After thorough evaluation, the number of HIV clients from the list that fulfills the inclusion criteria were 1228. A total of 489 Study participants were selected by using systematic random sampling method by which one random number in the Patient’s ART unique identification numbers as a starting point. The first HIV client was selected by lottery method among the first sampling intervals from the evaluated profiles (Fig. 1).

#### Data collection methods and instruments

A data collection tool was developed from ART entry and follow-up form being used in the ART clinic. The follow-up documents was evaluated thoroughly about its completeness before data collection takes place. The data was collected by reviewing medical record registers, laboratory requests, and follow-up form of ART. The data was collected from Jan 01–31/2014. Data quality was controlled through continuous supervision and random check-up of the data collection. Three days training was given for data collectors and supervisors. Data quality control during data entry was done by double entry to EPI-info Version 3.5.3 computer software and through multivariate analysis.

Ethical approval was obtained from the Institutional Health Research Ethics Review Committee (IHRERC) of Harar campus, Haramaya University, College of Medicine and Health Sciences. Following the approval by IHRERC, official letter of ethical clearance was written to the concerned bodies by the School of Public Health. As the study was conducted through review of medical records, the individual patients were not subjected to any harm and personal identifiers were not used on data collection forms and confidentiality was maintained.

#### Data processing and analysis

The data were entered into Epi-Info Version 3.5.3 and then exported to SPSS Version 16.0 and STATA version 12 statistical packages for data processing and analysis. Actuarial life table analysis was used to estimate cumulative proportion of surviving after initiation of ART. Kaplan–Meier Survival function was used to estimate mortality of HIV patients on ART. The log-rank test was conducted to compare time to death between/among various levels. Before running the Cox regression model, assumption of proportional-hazard was checked by Schoenfeld test and the assumption was not violated. Cox proportional-hazard regression was used to calculate the bivariate and adjusted hazard rate to determine independent determinants (P < 0.05) of time to death.

## Results

### Socio-demographic characteristics

The study involved a total of 489 adults of people living with HIV/AIDS (PLWHA) on ART; 163 (33.3%) were malnourished (BMI < 18.5 kg/m^2^) and 326 (66.7%) were non-malnourished adults (BMI ≥ 18.5 kg/m^2^). Most of the study subjects were females 254 (51.9%) and males 235 (48.1%). The overall mean(±SD) age at ART initiation was 34.36 ± 9.24 years, out of which most of them 201 (41.1%) were in the age range of 18–29 years followed by 149 (30.5%) of 30–39 years (Table 1).

**Table 1:** Socio-demographic characteristics of HIV-positive patients at ART initiation in Fiche Hospital, North Shoa, 2006–2013, (N = 489)

Characteristics	Malnourished (n = 163)	Non malnourished (n = 326)	Total (N = 489)	P value
	Number (%)	Number (%)	Number (%)	
Sex				
Male	95 (58.3)	140 (42.9)	235 (48.1)	0.002
Female	68 (41.7)	186 (57.1)	254 (51.9)	
Age groups (years)				
18–29	68 (41.7)	133 (40.8)	201 (41.1)	0.786
30–39	53 (32.5)	96 (29.4)	149 (30.5)	
40–49	31 (19.0)	69 (21.2)	100 (20.4)	
50+	11 (6.7)	28 (8.6)	39 (8.0)	
Marital status				
Single	24 (14.7)	37 (11.3)	61 (12.5)	0.139
Married	71 (43.6)	179 (54.9)	250 (51.1)	
Separated	21 (12.9)	43 (13.2)	64 (13.1)	
Divorced	22 (13.5)	31 (9.5)	53 (10.8)	
Widowed	25 (15.3)	36 (11.0)	61 (12.5)	
Educational status				
No education	60 (36.8)	78 (23.9)	138 (28.2)	0.004
Primary	57 (35.0)	107 (32.8)	164 (33.5)	
Secondary	36 (22.1)	116 (35.6)	152 (31.1)	
Tertiary	10 (6.1)	25 (7.7)	35 (7.2)	

P value <0.05 = statistically significant difference

### Baseline clinical and laboratory information of the cohort

The baseline mean (±SD) values for BMI of the participants was 19.75 ± 2.96. The median weight at ART initiation was 51 kg [interquartile range (IQR 45–57 kg)]. The median CD4 cell count at ART initiation was 145 cells/μl (IQR 80–222). Three hundred thirty five (68.5%) of the patients had CD4 counts <200 cells/μl. The median hemoglobin level was 12.90 g/dl (IQR 10.9–14.6). Most of the study subjects at ART initiation were 220 (45%) in WHO stage III and 197 (40.3%) in WHO stage II. With regard to functional status, 197 (40.3%) participants were ambulatory at baseline and 27 (5.5%) were bedridden. Out of those who participated, 304 (62.2%) had no previous opportunistic infection, 68 (13.9%) had one previous opportunistic infection and 117 (23.9%) had two and more previous opportunistic infections (Table 2).

**Table 2:** Baseline clinical characteristics of HIV patients in Fiche Hospital (N = 489)

Characteristics	Malnourished (n = 163)	Non malnourished (n = 326)	Total (N = 489)	P value
	Number (%)	Number (%)	Number (%)	
Functional status				
Working	49 (30.1)	216 (54.2)	265 (54.2)	0.0001
Ambulatory	94 (57.7)	103 (31.6)	197 (40.3)	
Bedridden	20 (12.3)	7 (2.1)	27 (5.5)	
WHO clinical stage				
Stage I	7 (4.3)	41 (12.6)	48 (9.8)	0.0001
Stage II	58 (35.6)	139 (42.6)	197 (40.3)	
Stage III	84 (51.5)	136 (41.7)	220 (45)	
Stage IV	14 (8.6)	10 (3.1)	24 (4.9)	
TB history				
Yes	75 (46)	73 (22.4)	148 (30.3)	0.0001
No	88 (54)	253 (77.6)	341 (69.7)	
Hemoglobin count (g/dl)				
<10	34 (21.5)	30 (9.7)	64 (13.7)	0.0001
≥10	124 (78.5)	279 (90.3)	403 (86.3)	
Previous OIs				
None	86 (52.8)	218 (66.9)	304 (62.2)	0.0001
One	18 (11.0)	50 (15.3)	68 (13.9)	
2+	59 (36.2)	58 (17.8)	117 (23.9)	

### Baseline demographic and clinical characteristics and associated mortality of patients on ART

From the study subjects, the proportion of mortality is higher among the age group of 50+ years followed by 40–49 years (20.5 vs. 19%). With regard to educational status, 36 (26.1%) of the participants who had no education died after initiation of ART. The proportion of mortality is higher among males than females (19 vs. 17%) (Table 3).

**Table 3:** Baseline demographic and clinical characteristics and associated mortality on ART in Fiche Hospital, Ethiopia

Characteristics	Total	Alive n = 402	Death n = 87	P value
	Number (%)	Number (%)	Number (%)	
Sex				
Male	221 (45.2)	179 (81)	42 (19)	0.604
Female	268 (54.8)	223 (83)	45 (17)	
Age groups (years)				
18–29	201 (41.1)	167 (83.1)	34 (16.9)	0.937
30–39	149 (30.5)	123 (82.5)	26 (17.5)	
40–49	100 (20.4)	81 (81)	19 (19)	
50+	39 (8.0)	31 (79.5)	8 (20.5)	
Nutritional status				
Malnourished	163 (33.3)	122 (74.8)	41 (25.2)	0.004
Non-malnourished	326 (66.7)	280 (85.9)	46 (14.1)	
Educational status				
No education	138 (28.2)	102 (73.9)	36 (26.1)	0.011
Primary	164 (33.5)	138 (84.1)	26 (15.9)	
Secondary	152 (31.1)	129 (84.9)	23 (15.1)	
Tertiary	35 (7.2)	33 (94.3)	2 (5.7)	
Functional status				
Working	265 (54.2)	257 (96.9)	8 (3.1)	0.0001
Ambulatory	197 (40.3)	128 (64.9)	69 (35.1)	
Bedridden	27 (5.5)	17 (62.9)	10 (37.1)	
WHO clinical stage				
Stage I	48 (9.8)	47 (97.9)	1 (2.1)	0.0001
Stage II	197 (40.3)	192 (97.5)	5 (2.5)	
Stage III	220 (45)	149 (67.7)	71 (32.3)	
Stage IV	24 (4.9)	14 (58.3)	10 (41.7)	
Previous OIs				
None	304 (62.2)	289 (95.1)	15 (4.9)	0.0001
One	68 (13.9)	50 (73.5)	18 (26.5)	
2+	117 (23.9)	63 (53.8)	54 (46.2)	
Initial ART regimen				
d4t(30)-3TC-NVP	83 (17)	72 (86.7)	11 (13.3)	0.245
d4t(30)-3TC-EFV	64 (13.1)	51 (79.6)	13 (20.4)	
AZT-3TC-NVP	230 (47)	193 (83.9)	37 (16.1)	
AZT-3TC-EFV	112 (22.9)	86 (76.8)	26 (23.2)	
HIV related counseling				
Yes	141 (28.8)	135 (95.7)	6 (4.3)	0.0001
No	348 (71.2)	267 (76.7)	81 (23.7)	
TB history				
Yes	148 (30.3)	101 (68.2)	47 (31.8)	0.0001
No	341 (69.7)	301 (88.2)	40 (11.8)	

### Survival analysis

A total of 489 HIV infected individuals were enrolled in a retrospective study for a median (IQR) of 22 (14–34) months; 17 (5–34) months among malnourished adults and 23 (17–34) months among non-malnourished adult patients. All the study subjects contributed 1545.4 person year of observation (PYO); 429.35 PYO for those who were malnourished HIV adult patients and 1116.05 PYO for non-malnourished patients. Out of the study subjects, 87 patients died during the study period giving a mortality rate of 5.63 per 100 person-year observations (87 deaths/1545.4 PYO). Of the 87 deaths, 27 (31%) occurred within the first 3 months of ART initiation and 41 (47.1%) died in the first year of follow-up. The overall estimated survival duration after ART initiation was 48 (95% CI 46.32–50.84) months.

**Fig. 2:**
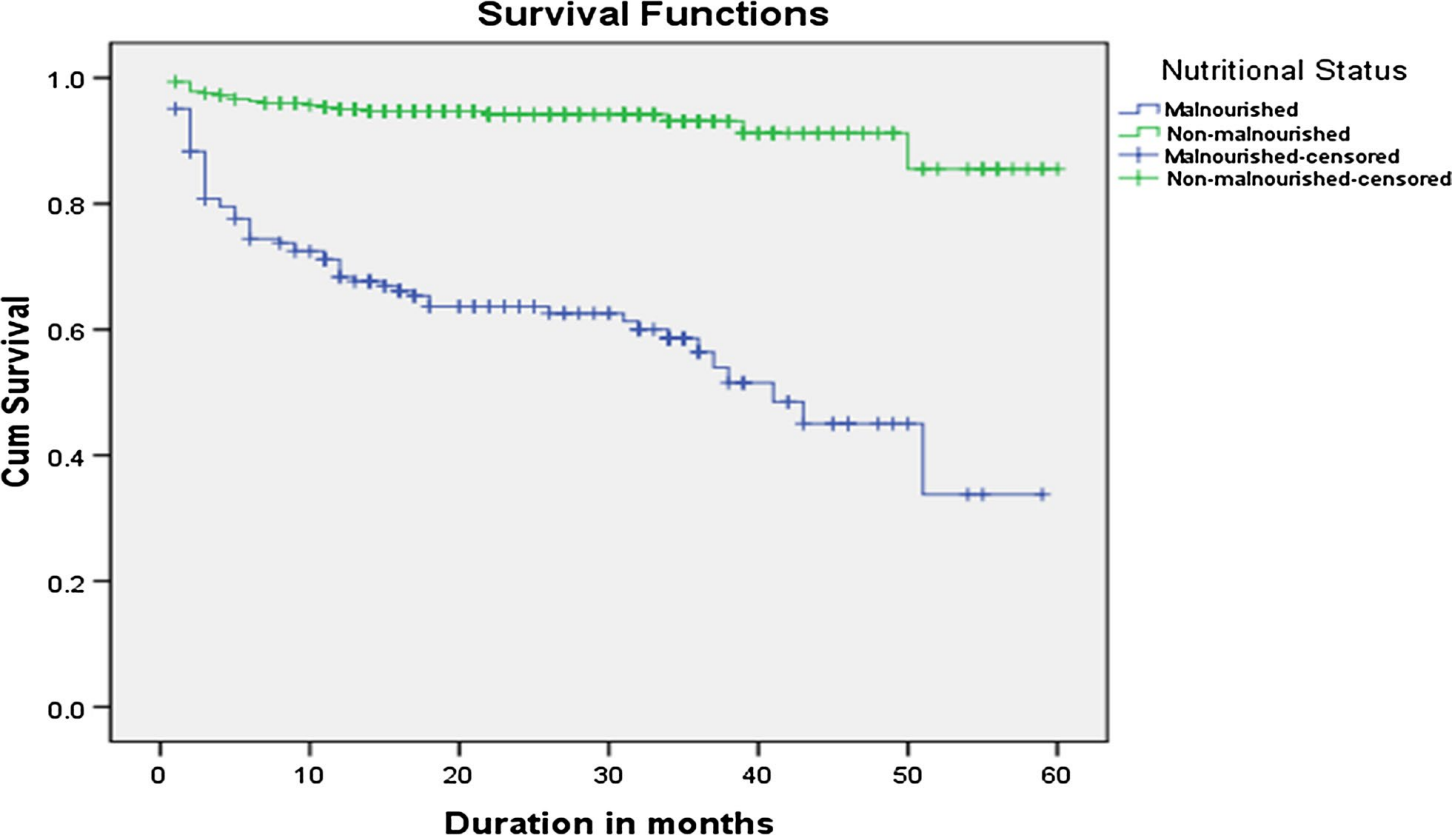
Survival graph of HIV patients at ART initiation by nutritional status in Fiche Hospital, Ethiopia

Actuarial life table analysis showed that probability of survival time among malnourished adult ART patients was 79, 91, 93, 94, and 98% at 5, 10, 15, 20, and 30 months respectively. The probability of survival time among non-malnourished adults was 97, 99, 99 and 98% at 5, 10, 15 and 35 months respectively (Fig. 2).

### Survival time of adult HIV-positive patients at ART initiation

#### Kaplan–Meier analysis of patients at ART by socio-demographic characteristics

The overall estimated survival time after ART initiation using Kaplan–Meier survival analysis was 48 months (95% CI 45.32–50.68). The survival experience after initiation of ART estimated by the Kaplan–Meier survival analysis by educational status showed that there was significant difference in median survival time among the groups, no education 44 months (95% CI 40.18–48.82) and secondary education 53 months (95% CI 49.98–56.02), (log rank test X^2^ = 14.321, P = 0.002).

#### Kaplan–Meier analysis of patients at ART by baseline clinical characteristics

There was significant difference in median survival time among working functional status 54 months (95% CI 52.49–57.51), ambulatory status 41 months (95% CI 36.88–44.22) and bedridden 34 months (95% CI 24.86–43.14) (log rank test X^2^ = 51.976, P = 0.0001).

### Predictors of mortality

After adjustment, the following characteristics at the initiation of the ART were the independent significant predictors of mortality: BMI < 18.5 kg/m^2^, baseline functional status (Ambulatory and Bedridden), WHO stage III and IV, CD4 cell count <200 cells/µl and opportunistic infections with two and more. Patients with a BMI <18 kg/m^2^ had a 5.4-fold increased risk of mortality (95% CI 3.03–9.58) as compared to those with ≥18.5 kg/m^2^. The hazard rate for dying is 3.84 times more with baseline functional status of ambulatory compared to patients with functional status of working (AHR = 3.84; 95% CI 2.19–6.74). Similarly, Patients with baseline functional status of bedridden had 4.78 times increased risk of death as compared to patients with baseline functional status of working (AHR = 4.78; 95% CI 2.14, 10.65). HIV-infected patients with baseline WHO clinical stage III had twofold increased risk of death compared to patients with stage I and II (AHR 2.21; 95% CI 1.16–4.21), and the risk of death among WHO clinical stage IV patients was even higher- compared to stage I or II patients (AHR 4.05; 95% CI 1.50, 10.97). Patients starting ART with CD4 count less than 200 cells/μl had threefold higher death hazard (95% CI 1.48–5.88) as compared to those starting ART with more than 200 cells/μl. Patients with two and above opportunistic infections had 2.3 times higher mortality as compared to those who had no starting opportunistic infection (AHR 2.30; 95% CI 1.11, 4.75) (Table 4).

**Table 4:** Bivariate and multivariate Cox-regression analysis of socio-demographic and baseline clinical characteristics of the cohort studied in Fiche Hospital, North Shoa during September 2006 to 2013, (N = 489 patients)

Covariates	Number at risk	Number of deaths	Crude HR (95% CI)	Adjusted HR (95% CI)
Educational status				
No education	138	36	1.86 (0.73, 4.76)	1.35 (0.50, 3.6)
Primary	164	26	1.60 (0.63, 4.10)	2.05 (0.78, 5.36)
Secondary	152	23	0.61 (0.22, 1.71)	0.92 (0.31, 2.74)
Tertiary	35	2	1	
Nutritional status				
Malnourished	163	41	7.58 (4.63, 12.39)	5.40 (3.03, 9.58)**
Non-malnourished	326	46	1	
Functional status				
Working	265	8	1	
Ambulatory	197	69	4.91 (2.91, 8.26)	3.84 (2.19, 6.74)**
Bedridden	27	10	6.86 (3.26, 14.44)	4.78 (2.14, 10.65)**
WHO clinical stage				
Stage I and II	245	6	1	
Stage III	220	71	3.1 (1.89, 5.07)	2.21 (1.16, 4.21)*
Stage IV	24	10	5.93 (2.89, 12.17)	4.05 (1.50, 10.97)**
TB history				
Yes	148	47	1.99 (1.31, 3.05)	0.95 (0.58, 1.55)
No	341	40	1	
CD4 count (cells/μl)				
≤200	335	260	3.44 (1.83, 6.48)	2.95 (1.48, 5.88)*
>200	154	142	1	
Hemoglobin level (n = 467)				
<10 g/dl	64	18	1.80 (1.06, 3.04)	0.82(0.45, 1.51)
≥10 g/dl	403	62	1	
Previous OIs				
None	304	15	1	
One	68	18	1.80 (0.99, 3.52)	1.31 (0.69, 2.5)
2+	117	54	3.24 (2.05, 5.13)	2.30 (1.11, 4.75)*
HIV related counseling				
Yes	141	6	1	
No	348	81	3.85 (2.44, 6.08)	0.57 (0.26, 1.24)

1.00 = Reference * P value <0.05, ** P value ≤0.001

## Discussion

Mortality in this study was found to be 5.63/100 person years at risk with most of the deaths occurred during the first 3 months following ART initiation. This result is in agreement with the study done in Burkina Faso [4]. However, this finding was low compared to common rates in resources-limited countries [5] and higher compared to 2.03/100 persons-years in the Eastern Ethiopia and 1.89/100 persons-years in Western Ethiopia respectively [6, 7]. The high early mortality observed in our study is in line with other similar studies from resource-limited settings, including Ethiopia [5, 8–12]. This may partly be explained by the fact that the majority of patients that 68.5% patients had advanced disease (CD4 count ≤200 cells/μl) and 45% patients had advanced clinical symptoms (WHO clinical stage III) at the time of treatment initiation and might be due to delayed diagnosis and/or treatment.

This study revealed that a low baseline body mass index (BMI) at the start of ART was an independent predictor of early mortality (i.e., in the first 90 days of therapy). This is in line with studies conducted in several sub-Saharan Africa [5, 11, 13–16]. This might be as a result of the aggregate effects of malnutrition-induced immune system dysfunction, a higher burden of opportunistic infections, metabolic derangement and anthropometric variations. Even after the initiation of ART, the side effects of certain antiretroviral drugs (e.g., nausea, insomnia) may prevent adequate intake [17], and malnutrition and low body weight may potentiate drug toxicity [18].

Patients with advanced clinical diseases (WHO stage III or IV) had higher mortality compared to patients with WHO stage I or II. This finding was supported by several other studies [11, 12, 19–22]. This might be due to the fact that patients died mostly because of their late initiation of ART when they had the worst health conditions. In contrast, a study conducted in Western Ethiopia and South Western Uganda reported that WHO clinical stage was not found to be associated with mortality [7, 23].

In this study, patients with two and above opportunistic infections had 2.3 times higher mortality as compared to those who had no starting opportunistic infection. This study established a similar finding with the studies in sub-Saharan Africa that showed OIs were found to be significant predictors of death among patients under ART [11, 13, 24, 25].

Adult HIV-infected patients who were bedridden at ART initiation had higher risk of mortality compared to the patients with working functional status at treatment initiation. This result is in line with the study done in Eastern Ethiopia and those described elsewhere [6, 21, 23, 26].

Patients starting ART treatment with CD4 cell count ≤200 cells/μl was an independent predictor of mortality in this study. This finding is consistent with studies [4, 6, 12, 20, 25, 27]. Studies have substantiated the fact that low CD4 cell count, a marker of advanced immunodeficiency, was associated with opportunistic infection thereby increasing the likelihood of death [28]. This may partly be explained by the fact that the majority of patients (75.5%) had a CD4 ≤200 cells/μl, which could have made the comparison with higher CD4 counts statistically unstable.

Our study was subjected to several important limitations. Selection bias is possibly introduced due to the fact that patients with incomplete records of variables were excluded. In addition, because we could not ascertain outcomes of patients lost to follow-up, our mortality results might be an underestimation. Anthropometric measurements might not be measured or recorded correctly.

## Conclusions

Undernutrition at the time of ART initiation was associated with increased risk of death, particularly during the first 3 months after ART initiation. With regard to nutritional status, there was a significant difference in median survival time between malnourished adults 35 months and non-malnourished adults 52 months. Being malnourished, late WHO stage, having low CD4 cell count, ambulatory and bedridden functional status and two and more opportunistic infections were factors independently associated with death. Interventions to promote earlier HIV diagnosis and treatment and nutrition counseling should be integrated at all stages of ART implementation, such as during adherence counseling, regular follow-up sessions, and meetings of PLWHA support groups may improve ART outcomes in this vulnerable population.

## Abbreviations

AHR: adjusted hazard rate
AIDS: acquired immunodeficiency syndrome
ART: antiretroviral therapy
BMI: body mass index
SRS: systematic random sampling
CHR: crude hazard rate
CI: confidence interval
Hgb: hemoglobin
HIV: human immunodeficiency virus
HR: hazard rate
IQR: inter quartile range
Kg: kilogram
OI: opportunistic infection
PLWHA: people living with HIV/AIDS
PYO: person-year observation
SD: standard deviation
TB: tuberculosis
WHO: World Health Organization
